# Tailoring magnetic properties of multicomponent layered structure via current annealing in FePd thin films

**DOI:** 10.1038/s41598-017-16963-5

**Published:** 2017-11-30

**Authors:** Matteo Cialone, Federica Celegato, Marco Coïsson, Gabriele Barrera, Gianluca Fiore, Ruslan Shvab, Uta Klement, Paola Rizzi, Paola Tiberto

**Affiliations:** 1 Chemistry Department and NIS, University of Torino,Via Pietro Giuria, 9-10125, Torino, Italy; 2 INRiM, Nanoscience and Materials Division, Strada delle Cacce, 91-10135, Torino, Italy; 3 Department of Industrial and Materials Science, Chalmers University of Technology, SE-412 96, Gothenburg, Sweden

## Abstract

Multicomponent layered systems with tailored magnetic properties were fabricated via current annealing from homogeneous Fe_67_Pd_33_ thin films, deposited via radio frequency sputtering on Si/SiO2 substrates from composite target. To promote spontaneous nano-structuring and phase separation, selected samples were subjected to current annealing in vacuum, with a controlled oxygen pressure, using various current densities for a fixed time and, as a consequence, different phases and microstructures were obtained. In particular, the formation of magnetite in different amount was observed beside other iron oxides and metallic phases. Microstructures and magnetic properties evolution as a function of annealing current were studied and interpreted with different techniques. Moreover, the temperature profile across the film thickness was modelled and its role in the selective oxidation of iron was analysed. Results show that is possible to topologically control the phases formation across the film thickness and simultaneously tailor the magnetic properties of the system.

## Introduction

Multilayered systems are nowadays of fundamental importance in manifold application, spanning from sensors to spintronic devices^1^. The assembly of those systems is performed using a number of different techniques such as sputtering^2,3^, pulsed laser deposition^4^, electrodeposition^5,6^, just to mention the most common. In this study, an innovative method for the synthesis of multicomponent layered system is presented. Thin films of FePd were sputtered and subsequently annealed using Joule heating. With this approach, which uses the heat generated by a current flow across the film, heating and cooling rates in the order of 10^4^–10^6^ K/s can be achieved^7^. The annealing process was performed in vacuum, with a controlled oxygen pressure, which promotes the preferential oxidation of iron. Consequently, a multicomponent system is obtained, in which different magnetic phases coexist in a well defined topological arrangement. Particularly interesting, for application in the field of spintronic, is the formation of magnetite (Fe_3_O_4_), a ferrimagnetic semiconductor^8^, inside the film. Indeed, magnetite displays a variety of spin transport effects such as spin Seebeck effect^9^, spin filter effect^10^ and spin valve effect in Fe_3_O_4_/MgO/Fe_3_O_4_^11^ junctions. In this framework, the combination of highly spin polarized materials, as spin sources, with semiconductors constitutes the prerequisites for spintronics devices such as spin-field effect transistor^12^. Therefore in this paper it is proposed an advanced technique capable to disclose new approach in the design and synthesis of systems with tailorable magnetic properties. The mechanism governing iron oxidation, the phase separation and the comparison of novel magnetic properties are here explained. Furthermore, an analytical model for the analysis of the temperature profile across the film thickness is proposed.

## Results and Discussion

### Structural, microstructural and compositional characterization

**Figure 1:**
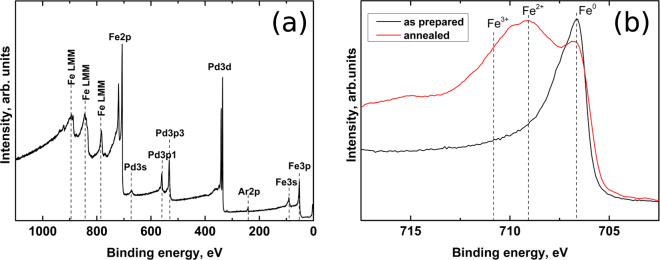
XPS spectrum: (**a**) Survey XPS spectrum for the as prepared film after Ar ions sputtering at a depth of 50 nm. (**b**) XPS spectra of the 2*p*_3/2_ for the as prepared and annealed at J = 1.63 · 10^8^ mA/cm^2^ films.

Composition of the as prepared samples was determined by means of different techniques: X-ray Photoelectrons Spectroscopy (XPS), Energy Dispersive X-ray Spectroscopy (EDS) associated with Scanning Electron Microscopy (SEM) and Transition Electron Microscopy (TEM). Results obtained were all consistent, showing a composition of 67 at.% Fe and 33 at.% Pd. XPS analysis confirmed that oxygen or other contaminants were not present in the as sputtered thin films surface. In order to check film composition and element distribution along the film thickness, XPS measurements were performed in a depth resolved configuration by etching the sample surface with Ar^+^ ions and an example is shown in Fig. 1(a), where the presence of Ar peaks is related to the etching process. A more accurate measurement was performed for the Fe 2p_3/2_ peak, in order to check for iron oxidation. As visible from the spectra of Fig. 1(b), in the as prepared samples iron is only present in a metallic state, while after annealing the presence of oxidation can be clearly inferred from the presence of Fe^3+^ and Fe^2+^ contributions. However, during depth resolved measurements, the etching conditions with Ar^+^ ions enable the reduction of Fe^3+^ to Fe^2+^, so that the [Fe^3+^]:[Fe^2+^] ratio obtained from the XPS spectra misrepresents the real Fe^3+^]:[Fe^2+^] ratio induced by the joule heating process. This effect was reported in literature, where a reduction of Fe_2_O_3_ to Fe_3_O_4_ by Ar^+^ sputtering is observed^13,14^.

**Figure 2:**
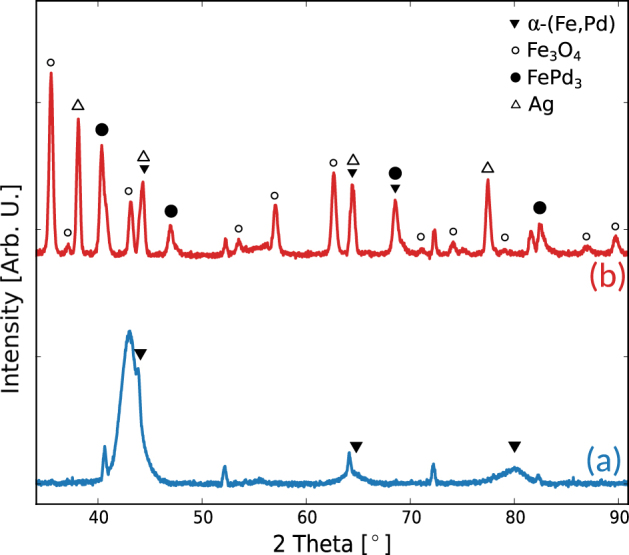
GIXRD patterns: for the (**a**) as prepared, and (**b**) annealed at J = 1.63 · 10^8^ mA/cm^2^ samples. The observed reflections of Ag are determined by the residual presence of silver paste used to establish the electrical contacts on the film surface. Unlabelled peaks belong to the substrate.

**Figure 3:**
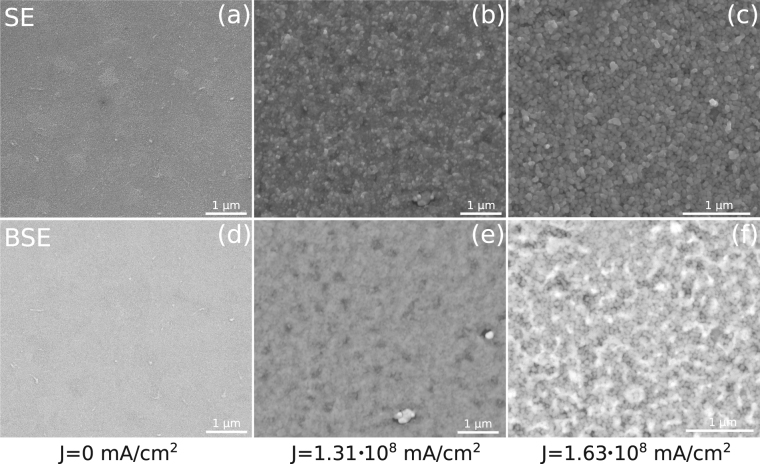
SEM images: (**a**), (**b**) and (**c**) are recorded using secondary electrons detector (SE). Images (**d**), (**e**) and (**f**) are recorded using back scattered electrons detector (BSE).

According to the stable Fe-Pd phase diagram, at room temperature, the presence of two phases (*α*-Fe + FePd) is expected for the Fe_67_Pd_33_ alloy. The x-ray diffraction pattern, taken at grazing incidence (GIXRD), of the as prepared samples (see Fig. 2(a)), however, shows, superimposed to the substrate peaks, the presence of broad reflections shifted to lower angles with respect to those due to pure *α*-Fe. This could be related to the formation, during sputtering, of a metastable supersaturated solid solution of *α*-(Fe,Pd) in which the Pd is non-uniformly distributed in the crystalline grains. Therefore, this concentration gradient produces diffraction peaks at slightly different angles, which overlap results in the broad reflections observed in Fig. 2(a). When samples are annealed by Joule heating treatments, an evolution of the metastable supersaturated solid solution is expected. In addition, the presence of oxygen species in the chamber induces selective oxidation of the film at high temperature. Indeed, the GIXRD pattern for the annealed sample, reported in Fig. 2(b), confirms a drastic change in the crystalline structure. The formation of iron oxides can be observed, alongside with the formation of a Pd-rich phase, which can be ascribed to a FePd_3_ phase. The oxidation process also affects the morphology of the film. The SEM images of Fig. 3(a) and (d) show the surface of the as prepared sample, which appears to be smooth and homogeneous. After the current annealing, as can be seen in Fig. 3(b) and (c), the roughness of the sample increases alongside with an increase in the grains size. Moreover, the formation of different phases, induced by the current annealing, can be observed in Fig. 3(e) and (f).

**Figure 4:**
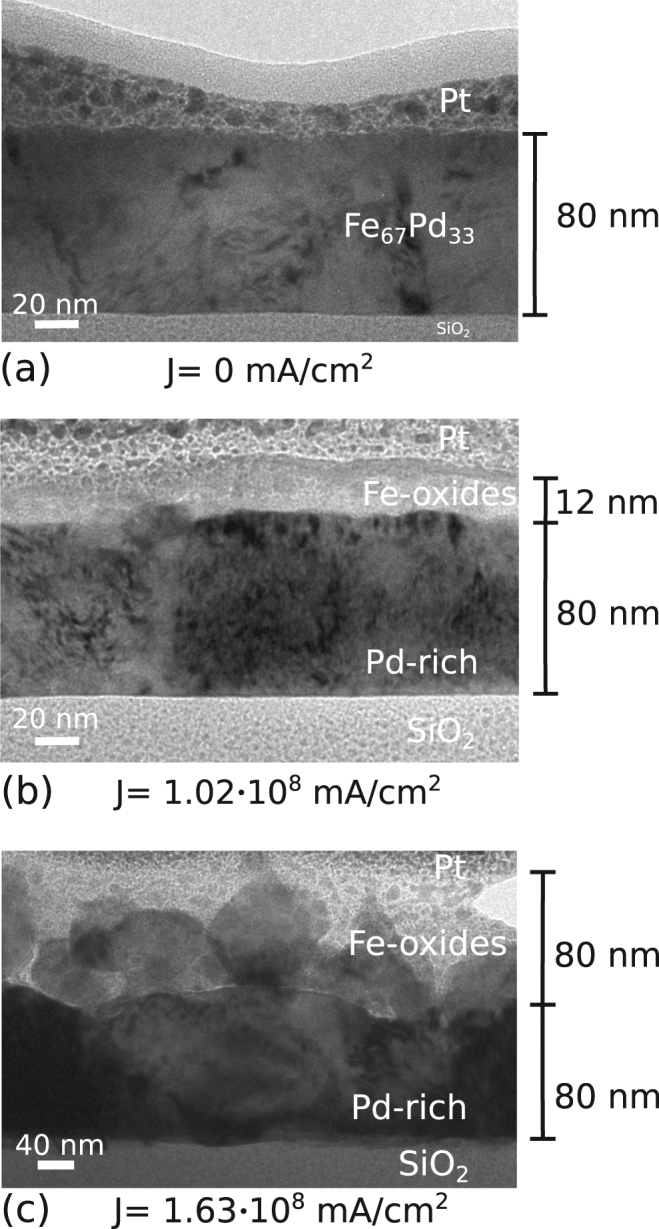
TEM images: different lamellas from samples annealed at selected current densities. The presence of an over-layer of Pt is determined by the lamella fabrication procedure.

In order to analyse the structure of the film across its thickness, thin lamellas of selected samples were prepared for TEM observation. The surface of the samples was covered with a platinum layer, in order to protect the surface during the fabrication process carried out using focused ion beam^15^. The micrograph in Fig. 4(a) shows the cross section of the as prepared film, which displays an homogeneous phase and a smooth surface. Whereas, in Fig. 4(b) and (c), the presence of different phases is observed in the annealed samples. Worth to notice that the current density was flowing in the film parallel to the interface with the substrate. More in details, by applying a low current density (J = 1.02 · 10^8^ mA/cm^2^), a thin layer of about 12 nm of Fe-oxides develops on top of the Pd-rich phase, whose composition, determined by TEM-EDS, is Fe_52_Pd_47_, while no Pd content is detect for the thin layer of oxide on top. By increasing the current density, the thickness of the iron oxide layer increases accordingly, reaching a maximum of 80 nm with the highest current density used (J = 1.63 · 10^8^ mA/cm^2^), as shown in Fig. 4(c). In this case, TEM-EDS analysis highlights a further increment of Pd content in the lower layer, whose stoichiometry results in Fe_36_Pd_64_. The increase of the overall thickness is related to the formation of FeO, Fe_2_O_3_ and Fe_3_O_4_ that are characterized by a misfit in volume with respect to the underlying metal. A measure of this misfit is given by the Pilling–Bedworth ratio^16^, defined as:

1 $$R=\frac{Molecular\,volume\,of\,oxide}{Molecular\,volume\,of\,metal}$$

Being R = 2.10 for Fe_3_O_4_/Fe, R = 2.14 for Fe_2_O_3_/Fe and R = 1.68 for FeO/Fe, the observed increase in thickness, as shown in Fig. 4, is consistent with the formation of Fe oxides. During current annealing, the Ellingham diagrams can predict that the aforementioned partial pressure of oxygen species, of the order of 1.0 · 10^−8^ mbar, could promote the formation of different iron oxides (FeO, Fe_2_O_3_ and Fe_3_O_4_)^17^. Moreover, the Joule heating chamber is kept at room temperature during the heat treatment so that water and oxygen molecules adsorbed on the FePd film are hardly removed from the surface and, therefore, an additional contribution to the formation of oxides can be expected with respect to a conventional heat treatment performed in vacuum in resistance furnaces. The mechanism acting during annealing can be suggested as follow: the presence of water and oxygen molecules adsorbed on the film surface induces the formation of Fe oxides starting from the Fe atoms present on the film surface and proceeding with the diffusion of Fe atoms from the bulk towards the surface. Taking into account the Ellingham diagram, and considering an isothermal annealing, the formation at first of a thick FeO layer is expected, being thermodynamically favoured, followed by the formation of a smaller amount of Fe_3_O_4_ and Fe_2_O_3_ on top of it, in form of layers^17^. The current annealing process, however, is non-isothermal, therefore, the formation of granular oxides structures could be envisaged; the sequence of oxides formation is dictated by the variation of the Gibbs free energy of Fe-oxides formation at high temperature, i.e. FeO, Fe_3_O_4_ and Fe_2_O_3_. As can be observed in the bright field TEM image of Fig. 4(b), the phase contrast of the Fe-oxide layer is uniform, therefore, the formation of a uniform layer of FeO can be inferred. Conversely, for higher current densities, the oxidation develops in the formation of different granular oxides, as can be observed in Fig. 4(c), in which different phase contrasts are evident. Therefore, the microstructure and hence, the magnetic properties of the multicomponent layered film can be tailor by tuning the intensity of the current density used in the annealing treatments.

In order to discuss the observed effect concerning the formation of Fe-oxides, it is convenient to investigate the temperature profile across a representative cross section of the sample during current annealing. However, given the complexity of the phenomena, a general case is here considered. Indeed, to correctly modelling the evolution induced by temperature changes, an atomistic approach is needed. Such a description is out of the scope of the present investigation. In our case a simplified procedure is followed, according with reference^18^. The Fourier equation of eat transfer is solved by considering the appropriate boundary conditions, at the film and substrate surfaces and at their interface. The result obtained from such analysis, shows that the position where the maximum temperature is achieved, lies at several hundred nanometers from the interface between the metal and the substrate. Therefore, the temperature in the film increases from the interface with the substrate towards the top surface, where radiation takes place.Rigorous derivation of the temperature profile across the film thickness can be found in the supplementary information section. This results can be directly related to the diffusion process of iron atoms. Indeed, according to Fick’s laws, interdiffusion takes place towards lower concentration and the diffusion coefficients increase with temperature. Concerning the self diffusion of Fe into Fe-Pd alloys, the diffusion coefficient are relatively high^18,19^, thus favouring the diffusion of Fe atoms towards the surface. The migration of iron atoms towards the film surface and their consequent oxidation increases the thickness of the superficial oxide layer. Furthermore, the iron diffusion determines the enrichment in Pd and the consequent phase transition from a BCC towards a FCC structure of the underlying layer, as shown in the XRD pattern of Fig. 2(b). Those arguments explain the morphology of the multilayered system, in which the different layers stratify parallel to the current flow across the film thickness.

### Magnetic characterizations

**Figure 5:**
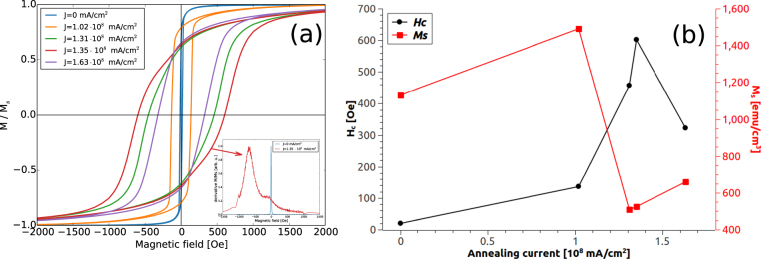
Magnetic characterizations: (**a**) hysteresis loops for the in plane direction of the magnetization measured at room temperature. In the inset: first derivatives of the blue curve, related to the as prepared film and red curve, related to current annealed film at J = 1.35 · 10^8^ mA/cm^2^. (**b**) Values of H_*c*_ and M_*s*_ for the different current densities.

Figure 5(a) shows the hysteresis loops at room temperature for the in plane component of the magnetization. The values of the coercive fields (H_*c*_) and saturation magnetization (M_*s*_) for the different samples, are extracted from the hysteresis loops of Fig. 5(a) and reported in Fig. 5(b). The hysteresis loops report the evolution of the magnetic properties of samples annealed with different current densities. For lower current densities (J ≤ 1.02 · 10^8^ mA/cm^2^), only the superficial iron undergoes oxidation, as can be seen in Fig. 4(b). The underlying portion of the film is partially enriched in Pd content, however the predominant phase is still the pristine metastable supersaturated solid solution of *α*-(Fe,Pd). At this stage, changes in the coercive field are determined by the crystalline grain coarsening. The portion of iron oxides increases on increasing current, determining a further enrichment in Pd for the underlying layer. The latter eventually gives the formation of a FePd_3_ structure, as indicated in Fig. 2(b). The FePd_3_ crystallizes in the L1_2_ phase with a FCC structure, which determines an increment of the crystalline anisotropy^20^, hence leading to an increase in the coercive field. However, this is not the only magnetic phase present in the system, since simultaneously, magnetite is formed. Indeed, considering the hysteresis loop of the film annealed at J = 1.35 · 10^8^ mA/cm^2^, shown in Fig. 5, it is visible a non monotonic change in the slope of the magnetization, indication of the presence of two different magnetic phases. To highlight this effect, first derivative curves of the magnetization, for the as prepared and for the annealed film, are reported in the inset of Fig. 5 (a). For the as prepared film, the derivative shows a peak centred at 19 Oe, corresponding to its coercive field. Conversely, for the sample annealed at J = 1.35 · 10^8^ mA/cm^2^, the derivative shows two peaks. The first sharp peak is centred at ≈690 Oe, corresponding to the coercive field of the predominant hard magnetic phase(i.e. FePd_3_). The second peak, centred at ≈100 Oe, is less intense and represents the soft magnetic phase (i.e. magnetite).

**Figure 6:**
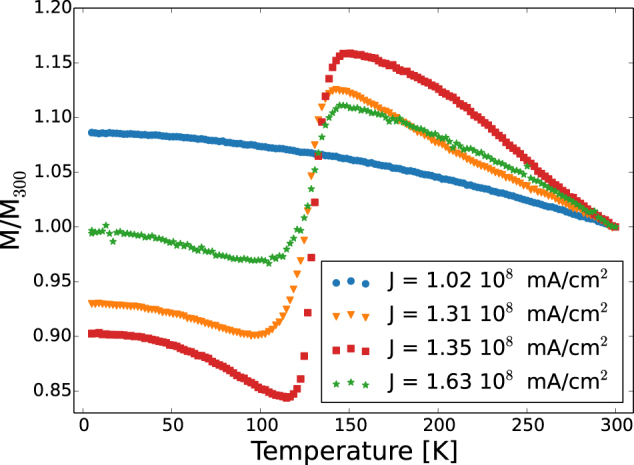
Magnetization as a function of temperature: M vs T curves for selected annealing currents densities.

To further investigate the role of the current on the oxidation of the iron, we measured the magnetization as a function of the temperature. Those measurement were performed with a superconducting quantum interference device (SQUID) for the in plane orientation of the magnetization. The samples have been previously demagnetized, subsequently the temperature was increased from 5 K to 300 K while applying an in plane constant magnetic filed of 300 Oe. For better visualization of the data, all the curves of Fig. 6 have been normalized to the magnetization at 300 K (M_300_). The magnetization of the sample annealed at J = 1.02 · 10^8^ mA/cm^2^ increases monotonically while reducing the temperature. Conversely, for the samples annealed with a current density higher than J = 1.02 · 10^8^ mA/cm^2^, a sharp jump of the magnetization is observed at around 120 K. This effect is the result of a structural transition typical of magnetite^21^, known as Verwey transition^22^. Increasing the current from J = 1.02 · 10^8^ mA/cm^2^ towards J = 1.35 · 10^8^ mA/cm^2^ a constant increase of the intensity of the jump of the magnetization can be observed. This can be directly related to an increase of the fraction of magnetite inside the sample, which strongly affect the overall magnetization of the sample. However, for the sample annealed with J = 1.63 · 10^8^ mA/cm^2^, the magnetization experiences a minor change, accounting for a reduce fraction of magnetite present in the sample. Hence, an increase of Fe_2_O_3_ and FeO phases, induced from the increasing in temperature, can be envisaged.

### Magnetic interactions

**Figure 7:**
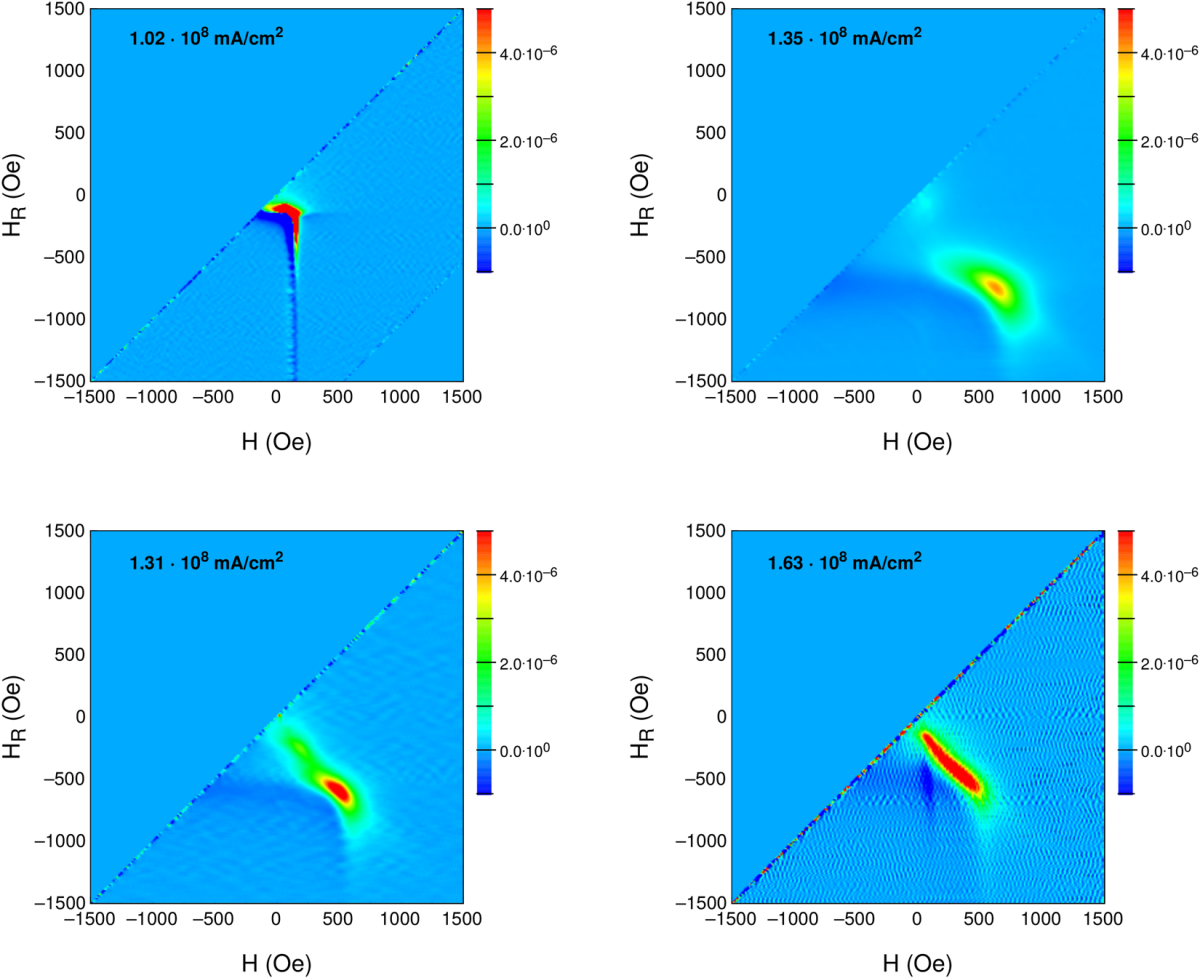
Magnetic interaction: Room temperature first order reversal curves for selected samples.

To investigate the interactions between the different magnetic phases in the films, first order reversal curves (FORC) have been collected at room temperature for the in-plane orientation of the magnetization. Subsequently, FORC diagrams have been elaborated^23^, as shown in Fig. 7. First order reversal curves represent the irreversible magnetization reversal process occurring in samples as a function of the applied field H and of the reversal field H_*r*_, starting from which the lower branch of the magnetization curve is measured after saturation^24^. Peaks in the FORC distribution identify irreversible magnetization reversal possibly arising from the presence of multiple, interacting magnetic phases. For the sample annealed at J = 1.02 · 10^8^ mA/cm^2^, a single peak is observed close to the origin, indicating a single soft magnetic phase reversing close to coercivity. In this case the soft magnetic phase is represented by the solid solution of *α* - (Fe,Pd), being the predominant phase present in the sample. Then, at increasing annealing current density, a new peak appears for H and H*r* values close to ≈600 Oe, indicating the development of a harder magnetic phase. As already pointed out, at this stage the system presents a layer of FePd_3_, which is responsible for the appearance of a peak at higher H_*r*_. However, the large halo towards the origin of the axes indicates that the softer phase is still present and interacting with the harder one. At the highest annealing current density (J = 1.63 · 10^8^ mA/cm^2^), the softer phase becomes more dominant but the broad FORC peak indicates a strong coupling between the two magnetic components. In this case, the formation of magnetite is evident, which determines the soft magnetic phase recognizable in the FORC diagrams. Interestingly, the amplitude in H and H_*r*_ of the FORC distribution is the same for all annealed samples, indicating that the two softer and harder magnetic phases remain approximately unchanged at all annealing current intensities; however their relative amount may change, and, above all, the coupling between the two phases is seen to evolve as a function of annealing, with an initial predominance of the role of the harder phase, with a subsequent predominance of the softer phase when the phase separation, indicated by micro structural analysis, is fully developed.

## Conclusions

In this paper, an innovative approach for the fabrication of multicomponent layered systems has been proposed and analysed. Starting from a homogeneous, single phase, thin film of Fe_67_Pd_33_ alloy, a multicomponent layered system has been obtained via post deposition current annealing. The current, flowing in the film, generates heat as a consequence of Joule effect. The modelled temperature profile across the film thickness shows that the film surface has an higher temperature as compared to the interface with the substrate. Iron atoms diffuse accordingly to the temperature gradient, thus enriching in palladium the lower section of the film. The residual presence of oxygen species in the chamber induces the formation of different iron oxides on the surface of the film. This process is depicted from the TEM images, which show the evolution of the aforementioned phases on increasing current density. Moreover, once a threshold current density is overcome, the formation of a magnetite phase has been observed. The presence of magnetite has been also proven via temperature dependent measurements of the magnetization, that shows a sharp jump at around 120 K, known as Verwey transition. Moreover, in order to investigate the interaction between the different magnetic phases at room temperature, first order reversal curves diagrams have been elaborated. Those diagrams unravel novel magnetic properties for the system, showing a non trivial interaction of the different magnetic phases.

## Methods

Fe_67_Pd_33_ thin films, with a thickness of 80 nm, were deposited onto Si/SiO_2_ substrates via radio frequency sputtering from a composite target. The composite target is obtained by addition of Pd tiles on top of a Fe target. The films were deposited with a constant rate of 0.15 nm/s. The base pressure of the sputtering chamber was 5.0 · 10^−6^ mbar, while during film deposition the Argon pressure was kept constant at 1.0 · 10^−2^ mbar. The deposition was performed at 50 W of power and the substrate was kept at room temperature. In order to induce oxidation of the Fe_67_Pd_33_ thin films, Joule heating treatments were performed in vacuum, with a base pressure of 1.0 · 10^−5^ mbar. The partial pressure of oxygen species in this conditions are in the order of 1.0 · 10^−8^ mbar^25^. The morphology and composition of the samples were studied by scanning electron microscopy (FEG-SEM) equipped with an energy dispersive x-ray spectrometer (EDS). Depth resolved x-ray photoelectrons spectroscopy (XPS) alternated with Ar^+^ ion etching^26,27^, was used to investigate films composition and element distribution in the film thickness. Structural information were obtained by means of grazing incidence x-ray diffraction (GIXRD). Magnetic characterizations of the samples were performed by means of different magnetometers, according to the investigated temperature range. Room temperature hysteresis loops for the in plane magnetization direction were obtained with an alternating gradient field magnetometer (AGFM). Low temperature measurements were taken using a superconducting quantum interference device (SQUID). To investigate the magnetic interaction between the different phases, first order reversal curves (FORC) were recorded at room temperature by AGFM^24^.

### Data Availability

The datasets generated during and/or analysed during the current study are available from the corresponding author on reasonable request.

## Electronic supplementary material

Supplementary information

## References

[1] Mills, D. & Bland, J. *Nanomagnetism, Volume 1* (Elsevier, 2006), 1 edn.

[2] Asti G (2006). Magnetic phase diagram and demagnetization processes in perpendicular exchange-spring multilayers. Phys. Rev. B.

[3] Nistor LE, Rodmacq B, Auffret S, Dieny B (2009). Pt/Co/oxide and oxide/Co/Pt electrodes for perpendicular magnetic tunnel junctions. Appl. Phys. Lett..

[4] Shen J, Gai Z, Kirschner J (2004). Growth and magnetism of metallic thin films and multilayers by pulsed-laser deposition. Surf. Sci. Rep..

[5] Péter, L. & Bakonyi, I. *Electrodeposition as a fabrication method of magnetic nanostructures* (World Scientific, 2012).

[6] Ross C (1994). Electrodeposited multilayer thin films. Annu. Rev. Mater. Sci..

[7] Zaluska A, Zaluski D, Petryk R, Zielinski PG, Matyja H (1985). Temperature distribution in D.C. Joule-Heated amorphous magnetic materials. Proc. Rapidly Quenched Met. 5.

[8] Fonin M, Dedkov YS, Pentcheva R, Rüdiger U, Güntherodt G (2007). Magnetite: a search for the half-metallic state. J. Phys.Condens. Matter.

[9] Ramos R (2013). Observation of the spin seebeck effect in epitaxial Fe_3_O_4_ thin films. Appl. Phys. Lett..

[10] Liao Z-Mo (2006). Spin-filter effect in magnetite nanowire. Nano Lett..

[11] Wu, H.-C., Mryasov, O. N., Abid, M. & K. R. Igor, V. S. Magnetization states of all-oxide spin valves controlled by charge-orbital ordering of coupled ferromagnets. *Sci. Rep*. **3**, 10.1038/srep01830 (2013). 10.1038/srep01830 PMC3652083 23665858

[12] Sugahara S, Tanaka M (2004). A spin metal–oxide–semiconductor field-effect transistor using half-metallic-ferromagnet contacts for the source and drain. Appl. Phys. Lett..

[13] Yamashita T, Hayes P (2008). Analysis of XPS spectra of Fe2+ and Fe3 +ions in oxide materials. Appl. Surf. Sci..

[14] P.Mills JS (1983). A study of the core level electrons in iron and its three oxides by means of x-ray photoelectron spectroscopy. J. Phys. D Appl. Phys.

[15] L.A. Giannuzzi, F. S. *Introduction To Focused Ion Beams* (Springer, 2005), 1 edn. Pp 201–28.

[16] McCafferty, E. *Introduction To Corrosion Science* (Springer, 2009), 1 edn. Pp 236.

[17] Young, D. J. *High Temperature Oxidation and Corrosion of Metals* (Elsevier, 2008), 1 edn. Pp 34–41.

[18] Aştefănoaei I, Radu D, Chiriac H (2005). Temperature distribution in D.C. joule-heated amorphous magnetic materials. J. Optoelectron. Adv. M..

[19] Yajun L, Jiang W, Yong D, Lijun Z, Dong L (2010). Mobilities and diffusivities in fcc Fe–X (Au, Cu, Pd and Pt) alloys. Calphad.

[20] E. Burzo PV (2010). Magnetic properties of iron-palladium solid solutions and compounds. J. Optoelectron. Adv. Mater..

[21] Iizumi M (1982). Structure of magnetite (Fe_3_O_4_) below the verwey transition temperature. Acta Crystallogr. Sect. B.

[22] Verwey EJW (1939). Electronic conduction of magnetite Fe_3_O_4_ and its transiotion point at low temperature. Nature.

[23] Pike CR (2003). First-order reversal-curve diagrams and reversible magnetization. Phys. Rev. B.

[24] Pike CR, Roberts AP, Verosub KL (1999). Characterizing interactions in fine magnetic particle systems using first order reversal curves. J. App. Phys..

[25] Umrath, W. *Fundamentals of vacuum technology* (Oerlikon Leybold vacuum, 2005), 1 edn.

[26] Shvab R, Hryha E, Nyborg L (2017). Surface chemistry of the titanium powder studied by xps using internal standard reference. Powder Metall..

[27] Hryha E, Shvab R, Bram M, Bitzer M, Nyborg L (2016). Surface chemical state of Ti powders and its alloys: Effect of storage conditions and alloy composition. Appl. Surf. Sci..

