# Supplementary material for: Tailoring magnetic properties of multicomponent layered structure via current annealing in FePd thin films
Source: Sci Rep. 2017 Nov 30;7:16691. doi: 10.1038/s41598-017-16963-5 (PMC5709423; doi:10.1038/s41598-017-16963-5)
Supplement: Supplementary file 1 — Supplementary information [file 41598_2017_16963_MOESM1_ESM.pdf]

Tailoring magnetic properties of multicomponent layered structure via current annealing in FePd thin films

Matteo Cialone[1,2,*], Federica Celegato[2], Marco Coïsson[2], Gabriele Barrera[2], Gianluca Fiore[1], Ruslan Shvab[3], Uta Klement[3], Paola Rizzi[1], and Paola Tiberto[2]

[1]Chemistry Department and NIS, University of Torino,Via Pietro Giuria, 9 - 10125, Torino, Italy
[2]INRiM, Nanoscience and Materials Division, Strada delle Cacce, 91 - 10135, Torino, Italy
[3]Department of Industrial and Materials Science, Chalmers University of Technology, SE-412 96, Gothenburg, Sweden
[*]matteo.cialone@unito.it

Supplementary information

Analytical model for the temperature profile across the film thickness

In order to discuss the observed effects concerning Fe-oxides formation in the current annealed films, it is convenient to investigate the temperature profile across a representative sample cross section during annealing. Following reference[1], the general procedure consists in solving the Fourier equation of heat transfer in the general case, considering the appropriate boundary conditions at the film and substrate surfaces, and at their interface. The general case, however, turns out to be an excessive overhead in our case, as the temperatures reached during annealing are so high that a remarkable evolution of the sample microstructure, and therefore of its resistivity as well, is obtained. Therefore, a simplified approach will be exploited here. A thin film of length L, width l, thickness g, on an electrically insulating substrate with the same lateral sizes and thickness G is considered. Let x be the coordinate along the sample length, y along its thickness and z along its width. Let y be zero at the interface between the film and the substrate. A cross section yz is considered that is far from the electrical contacts (e.g. approximately at half length of the sample), where a substantial independence of the thermal field on x can be assumed. Moreover, the temperature field it is assumed to be independent on z, i.e. the sample sides having thickness g do not contribute to heat dissipation. Therefore, the problem of the thermal conduction in the sample is simplified to a unidimensional case, y being the only coordinate along which temperature will vary. The Fourier heat conduction equation[1] can therefore be written as:

$$\frac{\partial^2 T(y)}{\partial y^2} + \frac{W_m}{\chi_m} = 0 \tag{S.1}$$

where the suffix m indicates that the relevant quantities refer to the metallic film, χ_m is its thermal conductivity and W_m is the sum of all the energy terms contributing to the heat generation and dissipation in the film. In particular, $W_m = W_{jh} + W_{rad}$. The Joule heating term is:

$$W_{jh} = \frac{\rho_0 I^2}{g^2 l^2} \tag{S.2}$$

where ρ_0 is the room temperature (initial) resistivity of the as-deposited film, I is the annealing current intensity and gl is the sample cross section. As the annealing current is continuous, and a homogeneous microstructure is assumed in the as-deposited sample, W_{jh} is independent on y.

The heat dissipation term due to irradiation is:

$$W_{rad} = \sigma \varepsilon_m L l \left(T(g)^4 - T_0^4 \right) \tag{S.3}$$

where σ is the Stefan-Boltzmann constant, ε_m is the radiance of the metal, T_0 is the room temperature, and $T(g)$ is the temperature at the top surface of the film (i.e. for $y = g$). There are no other heat dissipation mechanisms, since a cross section

Parameter	Value	Units	Notes
ρ_0	$1.3 \cdot 10^{-6}$	Ω m	representative value for as-deposited samples
I	$100-500$	mA	representative value for current annealing
g	100	nm	
l	3	mm	representative value
σ	$5.6704 \cdot 10^{-8}$	W m^{-2} K^{-4}	
ε_m	0.56	-	hypothetical value
L	15	mm	representative value
$T(g)$	1000	°C	hypothetical value
T_{sub}	200	°C	hypothetical value
χ_m	30	W K^{-1} m^{-1}	hypothetical value
G	0.5	mm	

Table S.8. Values of the quantities appearing in equation S.8.

far from the electrical contacts (that could drain heat) is considered, whereas convection at the pressures at which current annealing is performed only weights for a small fraction of the irradiated heat[1].

Equation (S.1) with the terms (S.2) and (S.3) has the general solution in the form:

$$T(y) = c_1 + c_2 y - \left[\frac{\rho I^2}{g^2 l^2 \chi_m} - \frac{\sigma \varepsilon_m L l \left(T(g)^4 - T_0^4 \right)}{\chi_m} \right] y^2 \tag{S.4}$$

where c_1 and c_2 are integration constants that will be obtained by properly imposing the continuity of the solution and of its first derivative at the interface between the film and the substrate[1].

In particular, the Si substrate will be characterized by a certain temperature gradient $\frac{\Delta T_{sub}}{G}$, ΔT_{sub} being the temperature difference between the two faces of the substrate (the interface with the metallic film and the opposite one). By imposing the continuity of $\frac{dT}{dy}$ at $y = 0$ (at the interface between the film and the substrate), one obtains:

$$c_2 = \frac{\Delta T_{sub}}{G} \tag{S.5}$$

Similarly, by imposing that the face of the substrate at the interface with the film has the same temperature $T(0)$ of the film, and by using equation (S.5), one obtains:

$$c_1 = T_{sub} + \Delta T_{sub}. \tag{S.6}$$

where T_{sub} is the temperature of the bottom surface of the substrate.

Under the stated conditions, the temperature profile of the film along its thickness is therefore given by:

$$T(y) = T_{sub} + \Delta T_{sub} + \frac{\Delta T_{sub}}{G} - \left[\frac{\rho_0 I^2}{g^2 l^2 \chi_m} - \frac{\sigma \varepsilon_m L l \left(T(g)^4 - T_0^4 \right)}{\chi_m} \right] y^2 \tag{S.7}$$

Since $T(y)$ has a parabolic behavior with downward concavity, the temperature has a maximum at a coordinate y_v obtained by imposing $\frac{dT}{dy} = 0$:

$$y_v = \frac{\Delta T_{sub}}{2G} \chi_m \frac{1}{\frac{\rho_0 I^2}{g^2 l^2} - \sigma \varepsilon_m L l \left(T(g)^4 - T_0^4 \right)} \tag{S.8}$$

Appropriate values for the quantities appearing in equation (S.8) are reported in table S.8. For several quantities, values are used that are representative of all the studied samples. For those quantities where hypothetical values are reported, they are intended to provide an order of magnitude of their values. As will be shown shortly, knowing them precisely is not necessary.

If the values reported in table S.8 are inserted in equation (S.8), it stands clearly that the radiative term is negligible with respect to the Joule heating one. Indeed, if this were not the case, the film surface would be cold, whereas as observed during current annealing, the surface becomes bright red because of its temperature increase. Equation (S.8) therefore simplifies into:

$$y_v = \frac{g^2 l^2}{\rho_0 I^2} \frac{\Delta T_{sub}}{2G} \chi_m \tag{S.9}$$

Even though the exact temperature difference between the two substrate surfaces is not known, it certainly amounts to several hundreds of degrees[2]. The y coordinate of the vertex of the parabola, i.e. the position where the maximum temperature is achieved, will therefore lie at several hundred, if not thousand, nanometers from the interface between the metal and the substrate. Therefore, $y_v > g$, indicating that the temperature in the film keeps on increasing from the interface with the substrate to the top surface, where radiation takes place.

If the substrate were extremely thick (G very large), with the same temperature difference ΔT_{sub}, then y_v would be much closer to the interface between the film and the substrate, as the temperature gradient across the latter would be much smaller. Instead, the large temperature difference between its two surfaces implies that the substrate is relatively efficient in draining heat from the metal film, in fact more efficient that the radiative losses on the top surface of the metal. Therefore, given the relatively small thickness of the studied films, their temperature increases from the interface with the substrate to their top surface.

It is important to remark that such analysis is qualitative because it relies on several assumptions. First, a stationary state is assumed, meaning that the same amount of heat is given to the system through current annealing and removed from it through dissipation. The transient to reach this stationary state could last longer than the annealing time; that, however, would only increase ΔT_{sub}, further supporting the conclusion that the top surface of the metal is hotter than the one in touch with the substrate. Then, no dependence of ρ_0 on temperature is taken into account. This is of course an approximation, justified by the fact that the temperatures reached by the metal during the current annealing are so high that significant structural transformations occur in the material. During these transformations, not only the resistivity changes, but as phase segregation occurs and oxides develop, the current distribution across the sample cross section will no longer be uniform. Taking into account all these complex details is beyond the scope of the present analysis, which only serves the purpose to assess a temperature gradient along the sample thickness that implies a top surface hotter than the interface with the substrate.

According to Fick's laws, interdiffusion takes place towards lower concentration and the diffusion coefficients increase with temperature. Concerning the self diffusion of Fe into Fe-Pd alloys, the diffusion coefficient are relatively high[1,3], thus favoring the diffusion of Fe atoms towards the surface. The migration of iron atoms towards the film surface and their consequent oxidation increases the thickness of the superficial iron oxide. Therefore, the iron diffusion determines the enrichment in Pd and the consequent phase transition from a BCC towards a FCC structure of the underlying layer, as shown in the GIXRD pattern.

Those arguments explain the typical morphology of the multilayered system, in which the different layers stratify parallel to the current flow across the film thickness.

References

1. Aştefănoaei, I., Radu, D. & Chiriac, H. Temperature distribution in D.C. joule-heated amorphous magnetic materials. *J. Optoelectron. Adv. M.* **22**, 933–950 (2005).

2. Coïsson, M. *et al.* Magnetic properties of current-annealed amorphous thin films. *J. Appl. Phys.* **112**, 933–950 (2012).

3. Yajun, L., Jiang, W., D.Yong, Lijun, Z. & Dong, L. Mobilities and diffusivities in fcc Fe–X (Au, Cu, Pd and Pt) alloys. *Calphad* **34**, 253 – 262 (2010).
